# EnsembleSeq: a workflow towards real-time, rapid, and simultaneous multi-kingdom-amplicon sequencing for holistic and resource-effective microbiome research at scale

**DOI:** 10.1128/spectrum.04150-23

**Published:** 2024-04-30

**Authors:** Sunil Nagpal, Sharmila S. Mande, Harish Hooda, Usha Dutta, Bhupesh Taneja

**Affiliations:** 1 CSIR-Institute of Genomics and Integrative Biology, New Delhi, India; 2 Academy of Scientific and Innovative Research (AcSIR), Ghaziabad, India; 3 TCS Research, Tata Consultancy Services Ltd, Pune, India; 4 Department of Gastroenterology, Post Graduate Institute of Medical Education and Research, Chandigarh, India; University of Arkansas for Medical Sciences, Little Rock, Arkansas, USA

**Keywords:** beyond bacteriome, mycobiome, nanopore sequencing, oral microbiome, multi-kingdom sequencing, amplicon sequencing, human microbiome, long-read sequencing

## Abstract

Bacterial communities are often concomitantly present with numerous microorganisms in the human body and other natural environments. Amplicon-based microbiome studies have generally paid skewed attention, that too at a rather shallow genus level resolution, to the highly abundant bacteriome, with interest now forking toward the other microorganisms, particularly fungi. Given the generally sparse abundance of other microbes in the total microbiome, simultaneous sequencing of amplicons targeting multiple microbial kingdoms could be possible even with full multiplexing. Guiding studies are currently needed for performing and monitoring multi-kingdom-amplicon sequencing and data capture at scale. Aiming to address these gaps, amplification of full-length bacterial 16S rRNA gene and entire fungal internal-transcribed spacer (ITS) region was performed for human saliva samples (*n* = 96, including negative and positive controls). Combined amplicon DNA libraries were prepared for nanopore sequencing using a major fraction of 16S molecules and a minor fraction of ITS amplicons. Sequencing was performed in a single run of an R10.4.1 flow cell employing the latest V14 chemistry. An approach for real-time monitoring of the species saturation using dynamic rarefaction was designed as a guiding determinant of optimal run time. Real-time saturation monitoring for both bacterial and fungal species enabled the completion of sequencing within 30 hours, utilizing less than 60% of the total nanopores. Approximately 5 million high quality (HQ) taxonomically assigned reads were generated (~4.2 million bacterial and 0.7 million fungal), providing a wider (beyond bacteriome) snapshot of human oral microbiota at species-level resolution. Among the more than 400 bacterial and 240 fungal species identified in the studied samples, the species of *Streptococcus* (e.g., *Streptococcus mitis* and *Streptococcus oralis*) and *Candida* (e.g., *Candida albicans* and *Candida tropicalis*) were observed to be the dominating microbes in the oral cavity, respectively. This conformed well with the previous reports of the human oral microbiota. EnsembleSeq provides a proof-of-concept toward the identification of both fungal and bacterial species simultaneously in a single fully multiplexed nanopore sequencing run in a time- and resource-effective manner. Details of this workflow, along with the associated codebase, are provided to enable large-scale application for a holistic species-level microbiome study.

**IMPORTANCE:**

Human microbiome is a sum total of a variety of microbial genomes (including bacteria, fungi, protists, viruses, etc.) present in and on the human body. Yet, a majority of amplicon-based microbiome studies have largely remained skewed toward bacteriome as an assumed proxy of the total microbiome, primarily at a shallow genus level. Cost, time, effort, data quality/management, and importantly lack of guiding studies often limit progress in the direction of moving beyond bacteriome. Here, EnsembleSeq presents a proof-of-concept toward concomitantly capturing multiple-kingdoms of microorganisms (bacteriome and mycobiome) in a fully multiplexed (96-sample) single run of long-read amplicon sequencing. In addition, the workflow captures dynamic tracking of species-level saturation in a time- and resource-effective manner.

## INTRODUCTION

The human microbiome represents the total gene pool of bacteria, fungi, viruses, protists, and other microscopic organisms inhabiting the human body (1). Bacteria (bacteriome) have traditionally received particular interest and, in fact, rather synonymously (mis)represented as the total “microbiome.” This is largely due to the historical focus on bacteria since the times of Leeuwenhoek (2), ease of bacterial culturing, an understanding that bacteria are the most abundant microbial cells in the human body, and a general lack of effective approaches to simultaneously study the total human microbiome (2, 3). The sparse population of other microorganisms, understood to constitute the dark matter of the human microbiome, largely remains to be (taxonomically and functionally) elucidated (3, 4). Recent reports of the human gut and oral mycobiome, especially the disorder-linked mycobiota (e.g., the pan-cancer fungi-typing), have now highlighted the understudied diversity and importance of the rare biosphere of the human body (5–7). Evidence is now accumulating toward not only the diagnostic potential of site-specific fungal communities in the human body but also their cross-talk with the dominating but co-inhabiting bacteriome (5–8). These strides for studying the mycobiome are encouraging and much needed for moving toward the holistic understanding of the (human) microbiome.

Amplicon sequencing has conventionally offered a cost-effective way of characterizing the human microbiome (9). Until the advent of long-read third-generation sequencing techniques (10), bacterial communities were profiled by amplifying and sequencing the short (~300–500 bp) segments pertaining to one or more of the variable regions (traversing V1–V9) of the ~1.5 kb long 16S rRNA gene (1, 9, 11, 12). The short segments of the variably long internal-transcribed spacer (ITS) region, (spanning ITS1, 5.8S, and ITS2) between the 18S and 28S rRNA genes, on similar lines served as universal barcodes for fungi (6, 8, 13, 14). Sequencing of the short segments of the marker genes, however, constrained the taxonomic classification primarily at the genus level, hence often limiting the majority of the (amplicon-based) human microbiota research to a rather under-resolved or shallow profiling (15, 16). Long-read sequencing technology has circumvented these limitations and enabled much resolved (species and even strain-level) characterization of microbial communities by targeting the full length of 16S and ITS regions (15–17). This advantage has reignited a deeper, species-level probing of the previously genus-level characterized human microbiota (15, 18, 19). At this juncture of revisiting the microbiome, skewed attention still remains toward the highly abundant bacteriome, with a limited number of independent studies reporting the characterization of human fungal microbiota using the entire ITS or large subunit regions (16–18, 20–28).

A holistic view of the microbial community of any environment must include information of the total microbiota (spanning different microbial kingdoms) to probe the role of the multiple prevalent microorganisms and their cross-talk (1, 3, 8). While this is possible through whole-metagenome sequencing, the cost of obtaining sufficient depth for a reliable assembly of all microbial genomes, overcoming the noise of rather dominant human or eukaryotic DNA, can be prohibitive, especially for large cohort studies (29, 30). It may be noted that amplicon sequencing can also turn expensive if (or amplicon type) separate sequencing runs and associated *in vitro* protocols are followed for every target kingdom. Multi-amplicon sequencing (i.e., combining the amplicon DNA from multiple genes or regions), usually employed for increasing the specificity of taxonomic profiling through assembly and consensus of multiple regions, can potentially also provide a resource-effective way of capturing different kingdoms of microorganisms in the same set of samples (12, 31).

The human microbiome is predominantly composed of bacterial cells (e.g., 10^11^–10^12^ CFU/g of cultivable bacteria in the feces or 10^9^–10^10^ CFU/ml of saliva in the oral cavity) (1, 32). A sparse content of other microorganisms, also called as the rare biosphere, is also observed in the human microflora (e.g., fungi, comparably forming 10^3^–10^6^ CFU/g of feces or 10^1^–10^4^ CFU/mL of saliva) (1, 4, 32, 33). This inherent sparsity of non-bacteriome populations in the total microbiome can perhaps offer an advantage in concomitantly sequencing a fraction of their (few kilobase long) marker amplicons (approximately 0.6 kb ITS region) along with the major proportion of the bacterial amplicons (approximately 1.5 kb 16S gene) without prejudicing the coverage of the either. This approach, here termed as “multi-kingdom-amplicon sequencing,” may potentially lower the time, effort, and cost of microbial community evaluation while also simultaneously characterizing the co-occurring endogenous residents of the human microbiota. A few recent reports, though using short reads and a small number of samples, have, in fact, provided encouraging evidence toward the potential to concomitantly capture both bacteriome and mycobiome using amplicon sequencing (31, 34). Current literature evidence, however, seems to indicate that a majority of non-bacteriome (primarily mycobiota) studies are done *in silos* (e.g., only mycobiome profiling of oral cavity) (5, 14, 20, 22, 35, 36) with a handful of short-read microbiome studies attempting both bacterial- and fungal-amplicon sequencing for the targeted samples, often as independent libraries or even independently sequenced bioprojects (31, 33, 37–39). Studies showcasing the success (and even failures) of the multi-kingdom-amplicon sequencing approach, with clearly outlined protocols, may add to the currently very limited evidence and encourage the (resource constrained) researchers to expand the scope of their microbiome research. This is also particularly important for the long-read technology and multiplexed experiments, which may demand high coverage or impact the sequence sufficiency, guiding case studies which are lacking.

**Fig 1:**
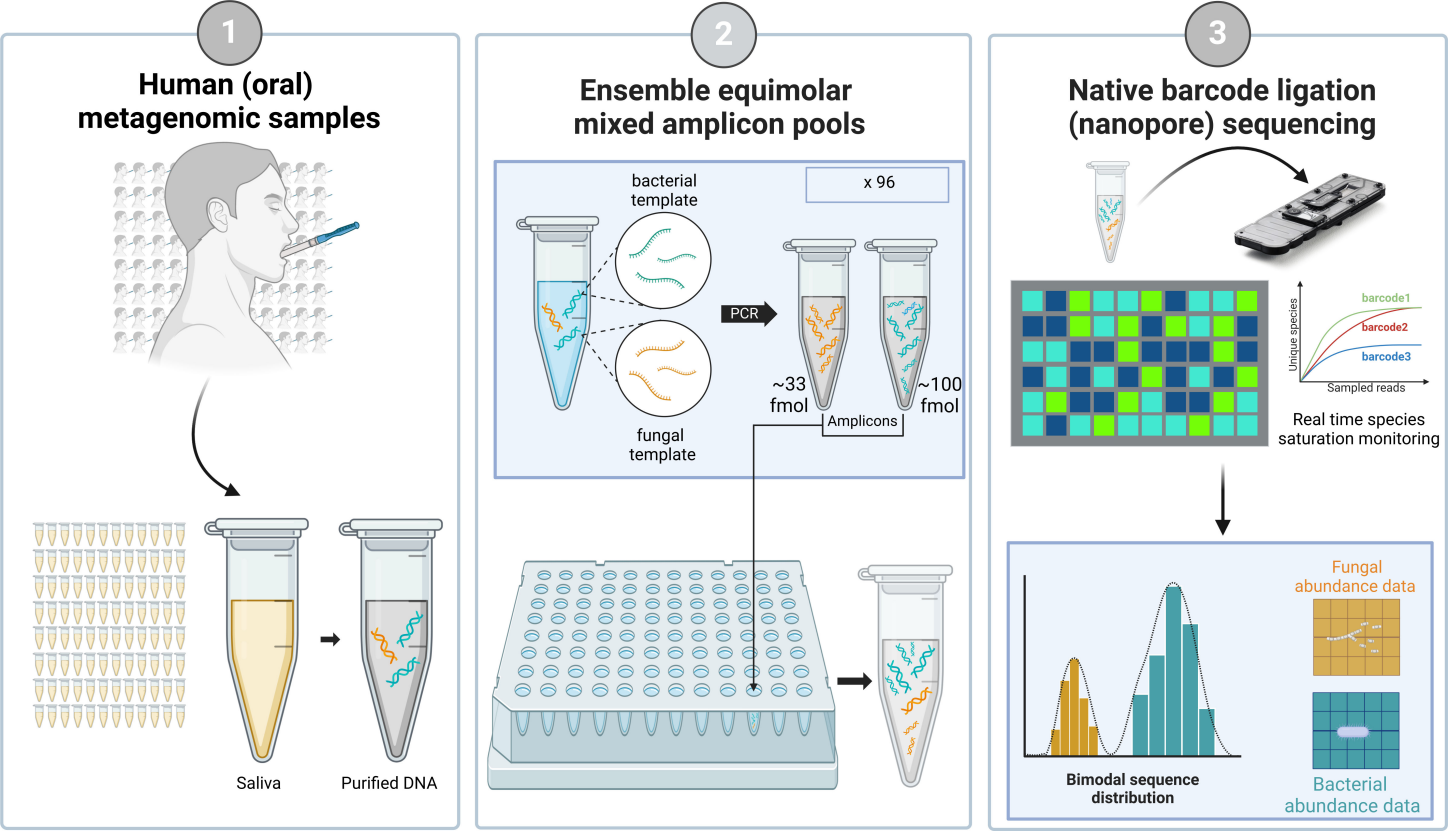
Graphical overview of the design of EmsembleSeq workflow.

**Fig 2:**
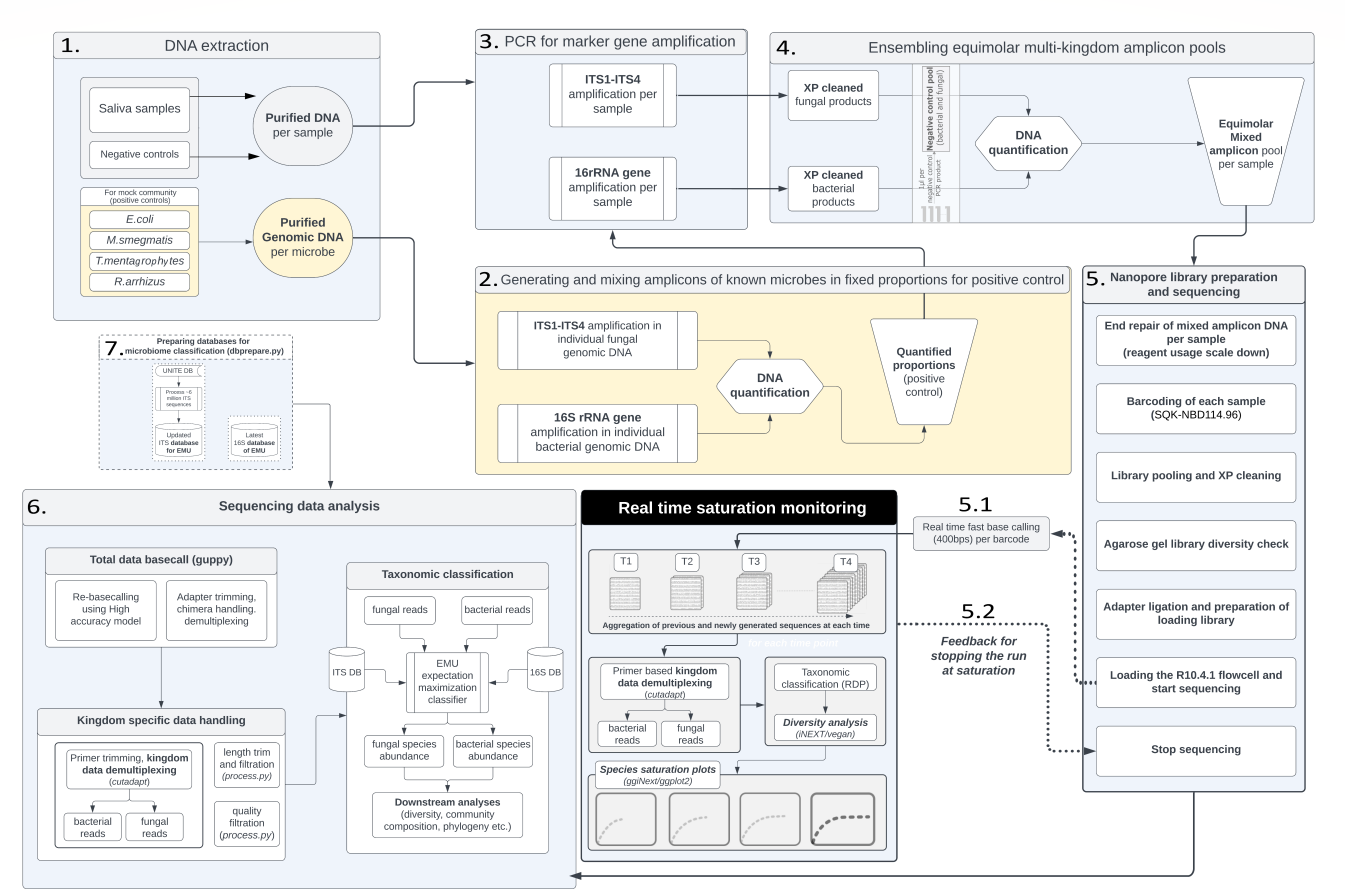
Complete workflow of EnsembleSeq providing schematic details of the various steps of both wet and dry lab experiments. These include DNA extraction with maintenance of negative and positive controls (steps 1–2), PCR for amplicon generation (step 3), negative control pooling and ensembling of multi-kingdom amplicons (step 4), mixed amplicon library preparation, nanopore sequencing and real-time saturation monitoring (step 5), sequence data analysis based on prior database preparation for microbiome classification (including species-level identification of bacterial and fungal communities, steps 6 and 7).

Here, as a part of a resource optimization effort for a larger microbiome cohort study in our lab, we present a guided workflow entitled “EnsembleSeq” (summarized in Fig. 1 and 2) as a strengthening proof-of-concept (first such case study using the nanopore technology) for a holistic multi-kingdom human oral-microbiome sequencing. Briefly, following avenues were explored that led to the design of EnsembleSeq: (i) attempting simultaneous (multi-kingdom) sequencing by spiking bacterial amplicons with a fraction of fungal amplicons, (ii) analyzing whether the spiking sufficiently captures the bacteriome as well as the mycobiome, (iii) devising an approach toward guiding the decisions for optimal run-time and timely decisions to avoid overutilization of the reusable flow cells, without impacting species saturation. Furthermore, the concordance of the concomitantly captured human oral bacteriome and mycobiome with the previous reports was also validated.

## MATERIALS AND METHODS

### Sample collection and DNA extraction

Human saliva samples were collected from 93 subjects, with dyspeptic symptoms, visiting the Department of Gastroenterology, Post Graduate Institute of Medical Education and Research, Chandigarh, India, upon informed consent after due approval of the designated Institutional Human Ethics Committee, in accordance with the guidelines of the Declaration of Helsinki, 1975 (IEC No. INT/IEC/2021/SP-809). Saliva was collected as a natural drool into 1.5 mL sterile eppendorftubes, mixed with RNAlater, and stored at −80°C.

DNA was extracted using DNeasy Blood and Tissue kit (QIAGEN, Germany) according to the manufacturer’s instructions, with additional steps of bead beating and lysozyme treatment. Briefly, 200 µL saliva sample was diluted in 1,000 µL tris-EDTA (TE) buffer (pH 8.0) in a 2 mL sterile screw cap vial (Biospec) and centrifuged at 10,000 g for 5 min. The pellet was resuspended in 190 µL TE and 10 µL (20 mg/mL) lysozyme, and the suspension was incubated at 37°C for 30 min. A mix of 0.1 mm and 1 mm sterile glass beads (Biospec) weighing 100 mg each was added to the vial, and homogenization was performed for 1 min using 200 µL disruptor genie, and the manufacturer’s instructions were followed for subsequent steps of DNA-extraction. The quality of purified DNA was assessed on a 1% (wt/vol) agarose gel, and quantification was performed using Qubit 3.0. Negative control (reagent only) samples were maintained in each batch of DNA extraction (Fig. 2, step 1).

### PCR amplification of 16S and ITS region

#### Amplicons for bacteriome

Given the advantage of full-length sequencing using Oxford Nanopore Technology (ONT), the entire 16S gene was targeted for amplification using the 27F (AGRGTTYGATYMTGGCTCAG) and 1492R (CGGYTACCTTGTTACGACTT) primers (18). PCR was set up in a set of two technical repeats for each sample as follows: 15 ng template, 0.3 µL (10 µM) 27F primer, 0.3 µL (10 µM) 1492R primer, 0.3 µL 100% DMSO, and 7.5 µL LongAmp Taq 2× ReadyMix (NEB) with volume made upto 15 µL with nuclease-free water. The PCR consisted of 1 cycle of denaturation (95°C, 5 min), 35 cycles of denaturation (95°C, 60 s), annealing (56°C, 30 s), and extension (65°C, 75 s), and 1 cycle of extension (65°C, 10 min). The technical repeat reactions were pooled for each sample.

##### Inclusion of positive and negative controls

One set of PCR reactions, as a positive control sample consisting of pre-quantified templates of 16S amplicons specific to routinely used laboratory strains *Mycobacterium smegmatis mc^2^155* (also, sometimes, known as *Mycolicibacterium smegmatis mc^2^155*) and *Escherichia coli DH5α* in 1:4 proportion, was also performed (Fig. 2, steps 1–3; File S1). This was done to facilitate a post-hoc analysis of potential PCR amplification and sequencing bias (if any). PCR was also similarly performed for negative controls.

All pools of amplicons were purified using a 1.8× Ampure XP clean-up as per the manufacturer’s instructions and eluted in 15 µL nuclease-free water. The quantification of the cleaned products was performed using Qubit 3.0.

### Amplicons for mycobiome

Fulllength ITS region was targeted through a semi-nested PCR using two sets of primers (39, 40) in the following set-up: Outer PCR (step 1): 30 ng template, 0.3 µL (10 µM) ITS1F primer (CTTGGTCATTTAGAGGAAGTAA), 0.3 µL (10 µM) ITS4 primer (TCCTCCGCTTATTGATATGC) (40, 41), 0.3 µL 100% DMSO, and 5 µL LongAmp Taq 2× Readymix with volume made upto 10 µL with nuclease-free water. The reaction consisted of 1 cycle of denaturation (95°C, 5 min), 35 cycles of denaturation (95°C, 60 s), annealing (56°C, 30 s), and extension (65°C, 60 s), and 1 cycle of extension (65°C, 10 min).

Inner PCR (step 2): 3 µL of step 1 product as template, 0.3 µL (10 µM) ITS1 primer (TCCGTAGGTGAACCTGCGG) (40, 41), 0.3 µL (10 µM) ITS4 primer (TCCTCCGCTTATTGATATGC), 0.3 µL 100% DMSO, and 7.5 µL LongAmp Taq 2× Readymix with volume made upto 15 µL with nuclease-free water. The reaction consisted of 1 cycle of denaturation (95°C, 5 min), 15 cycles of denaturation (95°C, 60 s), annealing (56°C, 30 s), and extension (65°C, 60 s), and 1 cycle of extension (65°C, 10 min).

#### Positive and negative controls

Similar to bacteriome, a positive control sample consisting of pre-quantified templates of ITS amplicons specific to clinical strains available in the lab, i.e., *Trichophyton interdigitale* UCMS-IGIB-CI1, belonging to *Trichophyton mentagrophytes/T. interdigitale* complex (GenBank Accession ID: OR899257) and *Rhizopus arrhizus* CAFI-CI-07 (GenBank Accession ID: OR889685), (in 2.7:1 proportion)*,* was also performed (Fig. 2, steps 1–3; File S1). PCR was similarly performed for negative controls. Nested amplicons were purified using a 1.8× Ampure XP cleaning as per the manufacturer’s instructions, eluted in 15 µL nuclease-free water, and quantified using Qubit 3.0.

One microlitre from all negative control cleaned products was pooled together to create a single “Negative Control Pool” (NCP; Fig. 2, step 4). This can help minimize the need for sequencing multiple negative controls when resources are limited, without neglecting the contaminants across different batches of upstream sample processing. The NCP and one randomly chosen negative control sample were selected for further processing and eventual sequencing. This resulted in a complete plate, i.e., a total of 96 samples (93 subjects, 2 negative controls, and 1 positive control) for the subsequent workflow.

### Native barcoding at scale, ensemble library preparation

ONT currently offers a single specialized-benchmarked kit, i.e., 16S barcoding kit (SQK-16S024) for microbiome research, focused only on bacteriome profiling (https://store.nanoporetech.com/16s-barcoding-kit-1-24.html). It allows a maximum of 24 unique samples in a single run using the proprietary-barcoded 16S primers, limiting the utility in studies aiming for scale in MINION devices. Given that 16S barcoding has been the default and benchmarked choice, many prior published reports of microbiome research using nanopore sequencing are often observed to be limited by scale in individual runs (15, 18, 42–44). In order to increase the scale of the captured microbiome, we sought to adopt the Native Barcoding Kit – 96 (SQK-NBD114.96) instead to natively barcode the sample-specific amplicons (total 96 samples) entailing end-repair/end-prep, PCR-free barcoding and adapter ligation. Furthermore, given our goal of concomitantly characterizing both the bacteriome and mycobiome of each sample, an ensemble approach was adopted. This method avoids the use of separate library preparation and flow cells for the sequencing of the 16S and ITS amplicons, thereby enabling a resource-effective multi-kingdom interrogation (described in Table 1; Fig. 2, step 4 File S1):

**TABLE 1:** Key steps and guiding principles of EnsembleSeq’s library preparation workflow

Step	Purpose	Recommended	Reagent quantities used	Guiding principle
Amplicon ensembling	Combining the multi-kingdom amplicons in independent equimolar proportions for unified library preparation and downstream ensemble sequencing	200 fmol of single amplicon product nuclease-free water (NFW) (volume upto 11.5 µL) DNA control (DCS): 1.00 µL	16S rRNA gene amplicons: 100 fmol ITS region amplicons: 33.3 fmol NFW (volume upto 7.67 µL) DNA control (DCS): 0.67 µL	Separate library preparation and subsequent sequencing runs for different kingdoms are time, effort, and resource intensive. 16S/ITS PCR products are a function of native microbial load. Small input DNA may only be available in site-/state-specific samples.
End repair	Preparing the ends of all 96-sample ensembled amplicon DNA molecules for subsequent full-scale barcoding	End-prep master mix: 2.5 µL (Ultra II Buffer: 1.75 µL, Enzyme: 0.75 µL)	End-prep master mix: 1.67 µL (Ultra II Buffer: 1.17 µL, Enzyme: 0.5 µL)	Third-party reagents incur high costs. Partial scale down of reagent usage may lower the investment of resources in large cohort studies.
Barcoding at scale	Full-scale multiplexing using 96 barcodes	End-prep DNA: 0.75 µL Barcode: 1.25 µL per barcode Blunt/TA ligase master mix: 5 µL	End-prep DNA: 0.75 µL Barcode: 1.25 µL per barcode Barcodes: 96 Blunt/TA ligase master mix: 5 µL	Microbiome studies often involve large cohorts and multiplexing. 16S barcoding kit (SQK-16S024) is specific to only bacteriome and multiplexes upto 24 samples. Use of native barcoding kit SQK-NBD114.96 enabled scope for large-scale study.
Adapter ligation	Ligation of V14 chemistry adapter sequences to the ends of the barcoded ensembled amplicon DNA to facilitate strand capture by the nanopores	Pooled barcoded sample: 30 µL Native Adapter (NA): 5 µL Quick ligation buffer: 10 µL Quick T4 DNA ligase: 5 µL	Pooled barcoded sample: 30 µL Native Adapter (NA): 5 µL Quick ligation buffer: 10 µL Quick T4 DNA ligase: 5 µL	V14 is the latest kit chemistry of ONT suitable for R10.4.1 flow cells with modal Q > 20, a better quality as compared to previous sequencing kits.

### Sequencing and real-time species saturation monitoring

The sequencing library was loaded into an R10.4.1 flow cell (FLO-MIN114) in an Mk1c machine running the MinKNOW (v 23.04.5). Fast basecalling (5 kHz 400 bps) with default Q **≥** 8 was enabled during sequencing for real-time access to the DNA sequences. For each barcode, the passed reads were subjected to real-time taxonomic classification adopting a read accumulation approach (Fig. 2, step 5; Fig. S1). Real-time taxonomic classification was performed for bacteriome and mycobiome using (fast approximate) ribosomal database project classifier (v2.14), and species saturation curves were dynamically plotted for each accumulated window using iNEXT (v3.0.0) and ggplot2 (v3.4.4) of the R programming language (refer Raredynamics in EnsembleSeq codebase). The sequencing was stopped when sequence generation started plateauing, and the majority of the samples were saturated in improvement for bacterial and fungal taxa identification between three pore-scans (especially for the samples with the least number of accumulated reads).

### High accuracy (re)base calling and sequence data preprocessing

After completion of the sequencing run, the raw signal (pod5) data were rebase-called (Fig. 2, step 6) using a high accuracy (HAC; 400 bps, 5 kHz) model of guppy-basecaller (v6.5.7+ca6d6afb8 with minimap2 v2.24-r1122) on a Windows OS based workstation (RAM: 64 gb, NVIDIA RTX A4000, 24 cores of 3.4 ghz processor). Sequences were demultiplexed as per the (96) barcode profile of the native barcoding kit. Adapters and barcodes were trimmed, and chimeric sequences were also disallowed through read splitting during the basecalling process using the following instructions for guppy (v6.5.7+ca6d6afb8):

guppy_basecaller.exe -i < PATH_raw> -s < PATH_store> -c dna_r10.4.1_e8.2_400bps_5 khz_hac.cfg -x 'auto' --recursive --do_read_splitting --barcode_kits SQK-NBD114-96 --detect_barcodes --enable_trim_barcodes

EnsembleSeq codebase further provides details of all the *in silico* steps.

The passed reads were subjected to primer trimming and segregation of bacterial and fungal reads using cutadapt (v4.4). Reads with a minimum read length of 200 and maximum length of 3,500 were retained. The quality profile of the passed reads was visualized using Nanoplot (v1.41.6) (45) and FastQC (v0.12.1) (46). Following the quality profile observed in FastQC for each position across the observed length of reads, read tails were trimmed to a final length of 1,550, and a further quality filtration of mean phred 20 was applied on the HAC-passed reads using the FastQC approach.

### Taxonomic classification

Taxonomic classification was performed at the CSIR-IGIB high-performance computing facility using Emu (v3.4.5) (15). It may also be noted that, while the latest database for bacterial classification was available, the fungal ITS database was outdated. A recent version of the fungal database for Emu was therefore created (Fig. 2, step 7) by employing more than 6 million full-length ITS sequences obtained from the latest release of UNITE ITS fasta database (accessed 18 July 2023) (47). The said database resource containing >48,000 unique ITS lineage entries, which can directly be adapted into Emu for mycobiota classification, is made available through the open science framework (OSF) at the following link: OSF | UNITE_million_db.

### Data analysis

Species saturation was analyzed through a rarefaction curve using vegan library (v2.6–4) of R programming language (48). Sample-level relative abundances of the top 10 phyla and species, with the highest number of cumulative reads in the study, were observed across all 96 samples (including negative and positive controls). The relative proportion of the top 10 genera was also analyzed. Data visualization was performed using ggplot2 (v3.4.4) (49) library of R. Cladograms representing the taxonomic lineage of the observed bacterial and fungal population, with at least 0.01% of relative proportion, were generated using Graphlan (v1.1.3) (50).

## RESULTS

### EnsembleSeq yields sufficient species saturation for the combinatorial microbiome in a time-efficient manner

**Fig 3:**
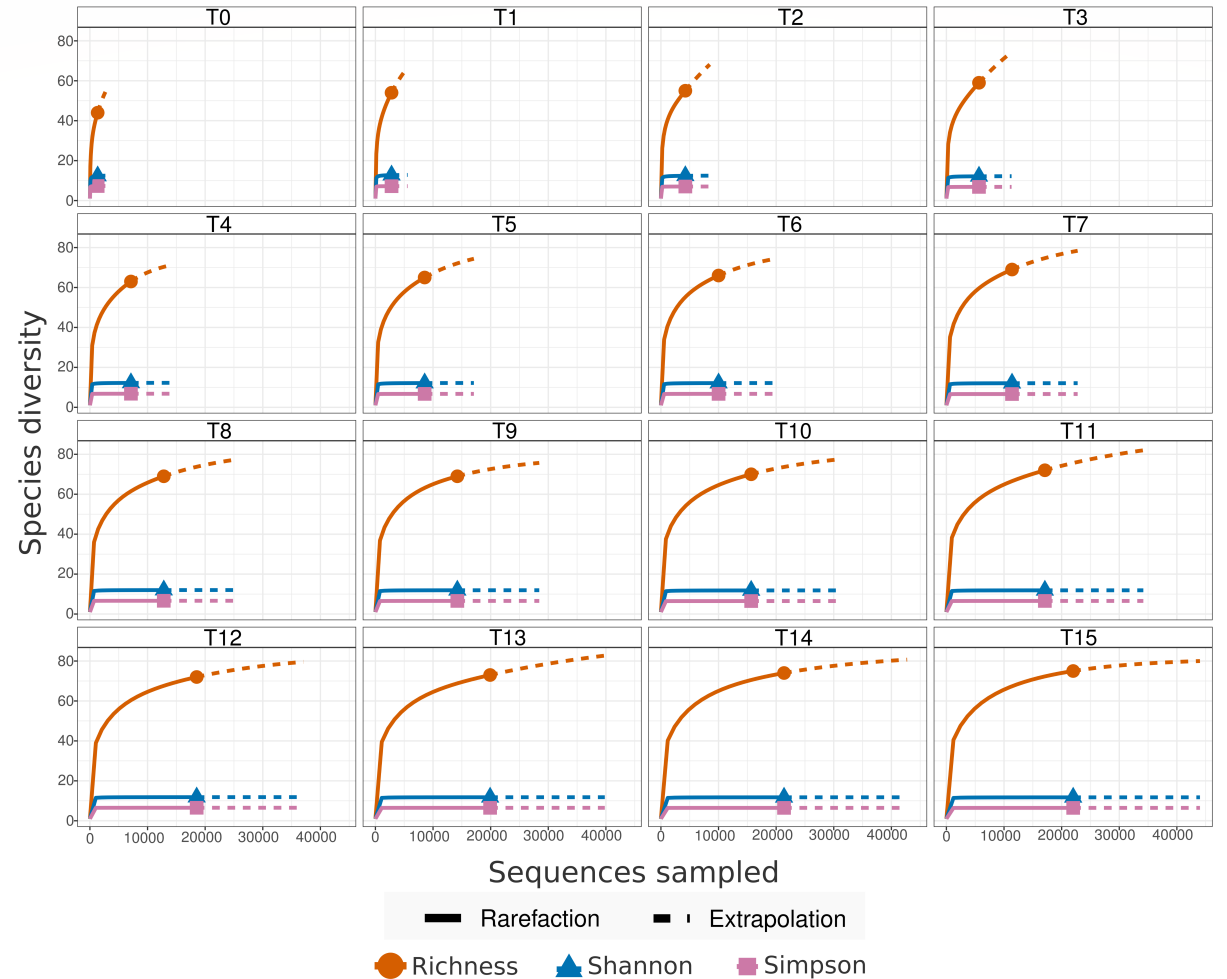
Real-time monitoring of bacterial species saturation in the sample specific to barcode1. T0–T15 indicate various time points of real-time sequence data generation with T0 referring to the first period of basecalling after initiating the sequencing. The run was configured to write 4,000 reads at a time.

EnsembleSeq helped concomitantly capture the major bacteriome as well as the sparse mycobiome in the studied samples without affecting the coverage of either. The real-time monitoring approach guided the timely decision for stopping the run upon species saturation in individual samples (Fig. 3), indicating the attainment of bacterial as well as fungal sequence sufficiency for the majority of the samples.

**Fig 4:**
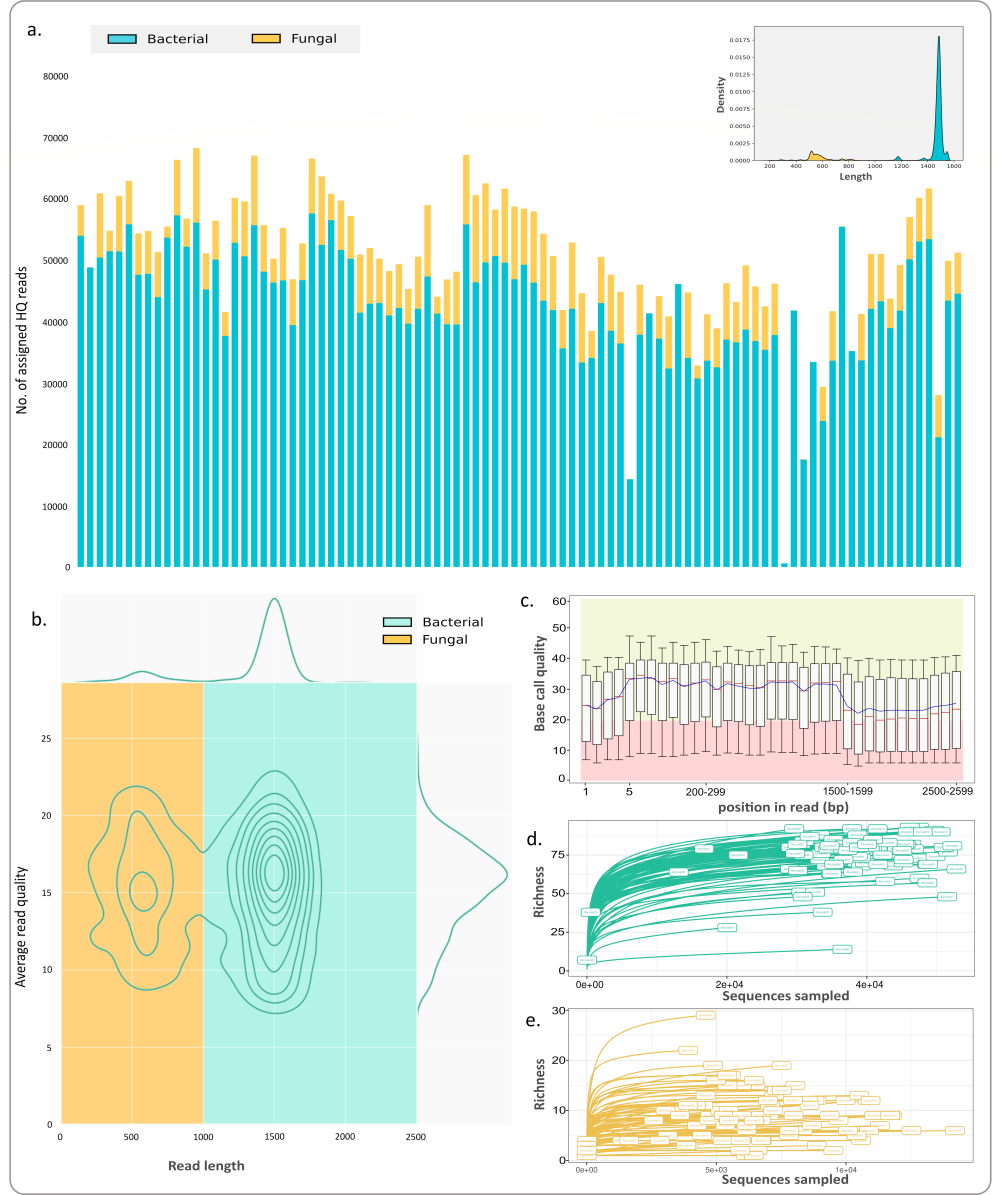
Summary of the data size, quality, and species saturation. (**a**) Number of high-quality assigned reads in the samples of this study. One outlier sample (barcode 55 with >240,000 reads) was omitted from this chart. Read length distribution for all the assigned reads in the study is presented in the inset. (**b**) Nanoplot generated summary of average read quality (>96% average read accuracy). (**c**) FastQC generated per base position quality trend (>99% modal accuracy per base call). (**d**) Species saturation curves for bacteriome. (**e**) Species saturation curves for mycobiome.

The sequencing run yielded a total population of 7.5 million raw reads with a median read accuracy of 95%. A total of 5.5 million reads passed the HAC basecalling, and 4.9 million (filtered) reads were used for taxonomic classification post length filtration and primer-based segregation of reads. Among these, 4.2 million reads pertained to bacteria, and 0.7 million pertained to fungi. The proportion of filtered reads (or library size) per sample was approximately uniform (Fig. 4a). The length distribution profile of the HAC passed, unfiltered reads exhibited an expected bimodal trend owing to a mix of fungal and bacterial sequences (Fig. 4b). Median read length for fungal reads was 556 bases while that of bacterial reads was 1,483 bases. The mean read quality for the filtered reads was >Q15 (>97% accuracy) for both fungal and bacterial reads as visualized in Nanoplot (Fig. 4a). The modal per basecall quality for each position of the reads was >Q20 (>99% accuracy) as visualized using FastQC (Fig. 4c).

Final species saturation post HAC re-basecalling also indicated sufficient capture of both fungal and bacterial diversity (Fig. 4d and e). Notably, the total run time was 30 hours against the recommended default of 72 hours. More than 40% of the nanopores (total 636) remained available for future re-use when the sequencing was stopped. EnsembleSeq hence enabled the collection of a large volume of high-quality data with species saturation for both the bacteriome and mycobiome in a combinatorial single sequencing run.

### Observed species-level taxonomic assignments for the oral microbiome

The combinatorial microbiome approach of EnsembleSeq enabled detailed taxonomic assignments for both bacteriome and mycobiome as described below.

### Top taxonomic units of the oral bacteriome

**Fig 5:**
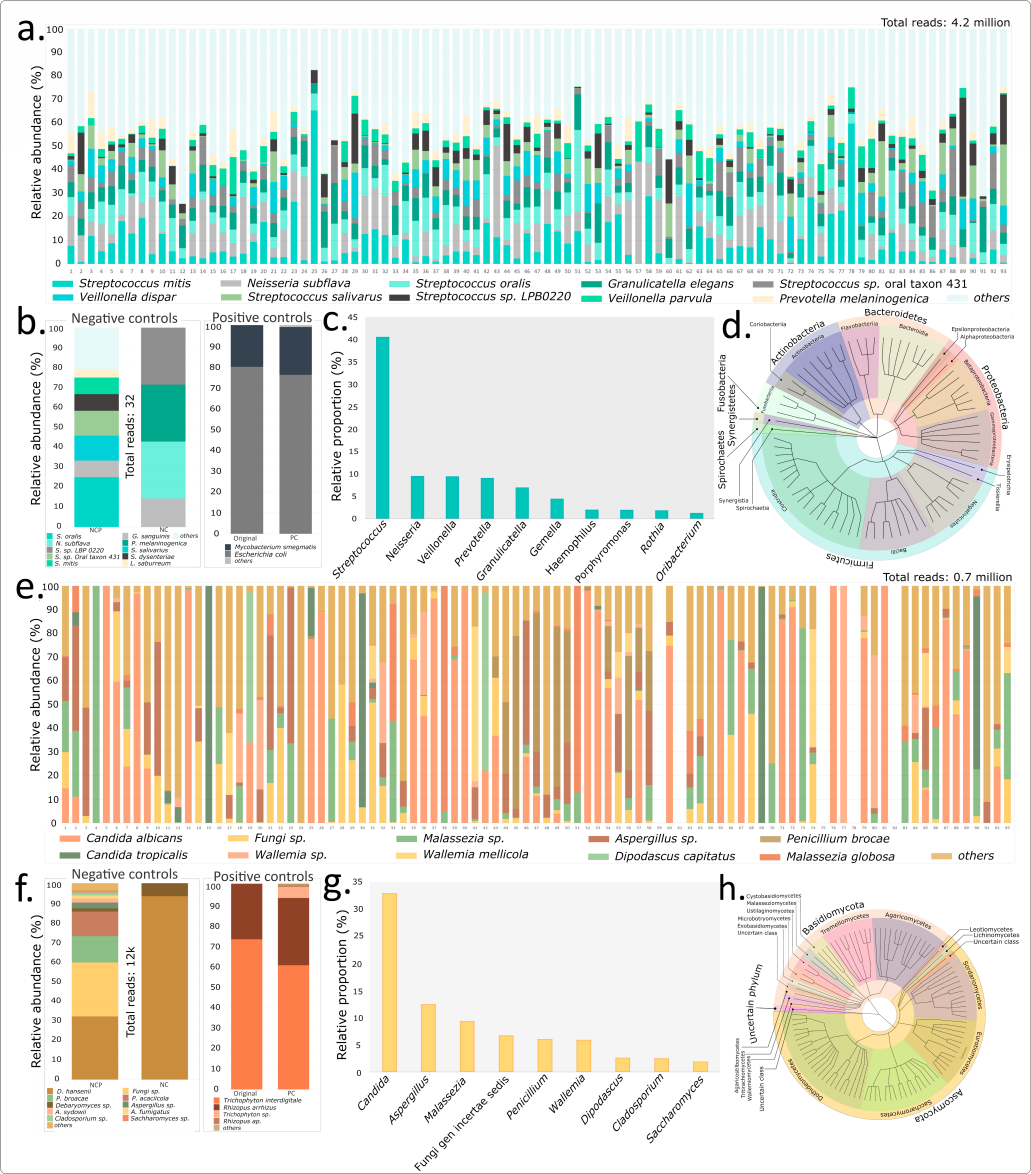
Relative abundance of high-quality assigned reads in the samples, their taxonomic specifications, and the affiliations for control samples of this study. (**a**) Species-level assignments of top 10 taxonomic units of bacteriome. (**b**) Control samples of bacteriome and proportions observed in the positive control set. (**c**) Top genus level assignment for bacteriome. (**d**) Cladogram depicting the taxonomic lineage highlighting phylum and class-level taxonomic affiliation of the bacterial genera with at least 0.01% of the assigned reads. Panels 5e-h represent the corresponding results for mycobiome.

The 4.2 million reads pertaining to bacteria were assigned to 401 species, affiliated to 125 genera and 5 major phyla namely Firmicutes, Proteobacteria, Fusobacteria, Bacteroidetes, and Actinobacteria (Figure S2a1; Fig. 5a through d). Five sparse phyla with a total of 5,701 reads assigned to them pertained to Spirochaetes (2547 reads), Candidatus Saccharibacteria (2306 reads), Synergistetes (768 reads), Tenericutes (55 reads), and Cyanobacteria (25 reads), and only 466 reads remained unassigned at any level of taxonomic hierarchy of bacterial kingdom. The classification results include the assignments for positive and negative controls (Figure S2a2).

Among the predominant phyla (Firmicutes, Proteobacteria, and Bacteroidetes) in the oral microbiota, the top three species-level affiliations pertained to *Streptococcus mitis*, *Neisseria subflava*, and *Streptococcus oralis* (Fig. 5a). At the genus level, 40.7% of the total reads were assigned to *Streptococcus*, followed by *Neisseria* (9.8%), *Veillonella* (9.6%), *Prevotella* (8.4%), *Granulicatella* (7.2%), *Gemella* (4.6%), *Haemophilus* (2.1%), *Porphyromonas* (2.1%), *Rothia* (1.9%), *Oribacterium* (1.3%), and *Stomatobaculum* (1%), among the top 10 assigned genera (Fig. 5c). The cladogram in Fig. 5d depicts the phylum- and class-level affiliation of all the identified bacterial genera in the study with at least 0.01% of assigned reads.

### Top taxonomic units of the oral mycobiome

The ~0.7 million reads of fungi on the other hand were assigned to 242 species of fungi, pertaining to 130 genera and 4 phyla (Ascomycota, Basidiomycota, Mucormycota, and one uncertain phylum), while 5,243 reads remained unassigned in total (Fig. S2b1). This included the assignments for positive and negative controls (Fig. S2b2).

The top two dominant fungal phyla across all samples of the study pertained to Ascomycota and Basidiomycota, along with an uncertain phylum affiliation observed across many samples (Fig. S2b1; Fig. 5e through h). *Candida albicans*, a frequently reported fungal member of the oral microbiota*,* was observed to be the dominant fungal species across majority of the samples, followed by *Malassezia sp.* and *Aspergillus sp*. among the other species-level designations (Fig. 5e). Among the total fungal reads, a majority (33.0%) was assigned to the *Candida* genus, followed by *Aspergillus* (12.6%), *Malassezia* (9.4%), uncertain genus (7.0%), *Penicillium* (6.2%), *Wallemia* (5.9%), *Dipodascus* (2.6%), *Cladosporium* (2.5%), *Saccharomyces* (1.9%), and *Buckleyzyma* (1.8%) as the top 10 genera with the most assigned reads (Fig. 5g). The cladogram in Fig. 5h depicts the phylum- and class-level affiliation of all the identified fungal genera in the study with at least 0.01% of the assigned reads. Mucormycota with less than 0.01% obtained reads is likely not a key fungal phylum of the oral microbiota.

The observed community composition of the bacteria and fungi conformed well with the existing evidence pertaining to the human oral microbiota, as detailed in the Discussion section.

### Analysis of control samples

Positive (PC) and negative (NC) control samples were included to assess any bias in amplification, sequencing, and assignment. Sufficient saturation was obtained for both the bacteria and fungal PC. The obtained reads were subjected to the defined workflow for confirming species-level classification, as carried out for the oral microbiome samples. Thirty-four thousand seven hundred eighteen and 10,423 reads could be assigned, respectively, to *E. coli* and *M. smegmatis* correctly upto the species level, indicating a close species-level conformity (1:3.33) with the originally input proportion (1:4) for *E. coli* and *M. smegmatis* (Fig. 5b).

Similar to bacteria, a close conformity was observed between the relative proportions of the originally input proportion of *T. interdigitale and R. arrhizus* (2.7:1) and the observed fungal species proportions (6,145:3,327 reads or 1.85:1) in ensemble sequencing of the positive controls (Fig. 5f). Accounting for assignments pertaining to *Trichophyton sp*. (546 reads) and *Rhizopus sp*. (59 reads) provided a final ratio of 1.98:1, suggesting good concordance with the input proportions and hence a limited bias due to PCR, library preparation, sequencing, or taxonomic classification in the overall workflow.

Negative control samples were primarily observed to be devoid of bacterial contamination (Fig. 5b). The fungal reads in these controls primarily pertained to *Debaromyces hansenii*, which was not found as a dominant native member of the oral microbiota samples (Fig. 5f). Sparse content of *Penicillium brocae*, *Aspergillus sp*., *uncertain fungus*, and *Cladosporium sp*. reads was also present in the negative controls (Fig. 5f).

## DISCUSSION

Multi-kingdom-amplicon sequencing, especially using the state-of-the-art (long read, real-time) Oxford Nanopore Technology, holds promise in the low-cost, holistic, and species-level characterization of the (human) microbiota. Here, we attempted a proof-of-concept in concomitantly profiling the oral human bacteriome and mycobiome starting right from the library preparation to a fully multiplexed (96-sample) single sequencing run using MinION (ONT).

Motivated by the need for resource optimization, this proof-of-concept study offered a rational advantage of lower resource utilization by combining multiple amplicons for unified library preparation and sequencing. We discuss in Table 2, various directly quantifiable resource advantages of the proposed workflow, highlighting the advantages of EnsembleSeq-like designs in resource-effective multi-kingdom-amplicon sequencing.

**TABLE 2:** Summary of quantifiable resource effectiveness in the use of EnsembleSeq

Workflow component	Original Resources needed*^a^*	EnsembleSeq-guided usage (this study)	Guiding principle
Ensembling of amplicons	One set of all reagents and apparatus for each amplicon type	1/A (50%)	Microbiome study workflow is multi-modal and complex. Going beyond bacteriome would require either a whole-metagenome study or additional sequencing of kingdom-specific amplicons. Unless combined or ensembled, a proportional amount of resources and time are needed for characterizing every intended amplicon type. Independent runs can additionally incur batch effects and associated errors.
Scaling down of input DNA and reagent usage	100% of the protocol recommended quantity of input DNA and end-repair reagents	2/3 (66.67%)	Multiplexed libraries are pooled prior to sequencing. The need for large input DNA per sample can hence be avoided in fully multiplexed designs. A large amount of input DNA would also require an optimized large reaction size of PCR or multiple technical repeats. Microbiome samples, especially with low microbial load, may not necessarily yield the needed amounts of input DNA. The reagents and enzymes employed in end-repair also incur high costs. Scale-down of input DNA and proportional reduction in the end-repair reaction volume may maintain the recommended concentrations while lowering resource usage.
Flow cells for sequencing	One for each amplicon type	1/A (50%)	Independent runs for each target kingdom usually require at least one flow cell for each kingdom type (without reuse; e.g., one flow cell each for bacteriome and mycobiome). Ensembling reduces the use of flow cells and hence associated cost, such that the more the number of target amplicons are combined, the lesser the cost or flow cell usage. Notably, a whole-metagenome sequencing run can be flow cell and resource intensive. For example, in a recent work by reference (51), the author used three flow cells for the whole-genome sequencing (WGS) run for each of the three metagenomic (saliva) samples in the study. EnsembleSeq leverages low data needs of amplicon sequencing, demonstrating a fully multiplexed (96 samples) run in a single flow cell, allowing concomitant characterization of both bacteriome and mycobiome.
Saturation monitoring	72 hours recommended run time and one complete flow cell	Depends on library quality and sample complexity (30 hours, 60% of pores)	ONT flow cells can be reused provided there are sufficient nanopores available. Optimal usage of the flow cell and timely decisions on stopping the runs can ensure the availability and recovery of nanopores for reuse of the flow cell. Species saturation monitoring can help estimate whether there is merit in continuing the sequencing, thereby aiding the decision-making for optimal run time. This can optimize the resources (flow cell), time, as well as data management (amount of data generated).
NCP	One set of all reagents and apparatus for each negative control sample	2/N (~17%)^*b*^	Resource constraints may limit the ability to use all negative controls of the study for sequencing [requires slots (often max 96 wells) in the library preparation kit]. Separate sequencing runs may be needed to accommodate all clinical samples as well as negative controls.

^*a*^ Starting from library preparation; **N** is the total negative controls in the study (e.g., 12 negative controls were maintained in the current study); **A** is the total number of amplicon types (e.g., two amplicon types, i.e., 16S and ITS were employed in this study).

^*b*^ EnsembleSeq used two negative controls—one NCP and one random batch-specific negative control (NC), hence a resource requirement of 2 /N.

In the absence of prior guiding reports for long-read multi-kingdom-amplicon sequencing, the key priors (e.g., ideal proportions of mixed amplicons) in our study were guided by the inherent sparsity of the non-bacteriome populations and the need for sufficient coverage for at least the highly abundant and diverse bacterial microflora. We, hence, sought to spike a minor fraction of the full-length ITS region (fungal) amplicons (~33 fmol) to the major proportion of the full-length 16S rRNA gene (bacterial) amplicons (~100 fmol). The said proportions of amplicons were mixed with an anticipation that the minor fraction should not be too low for the sparsely abundant fungi, and the remaining fraction should not undercover the native bacterial community intended to be captured through the full-length 16S rRNA gene sequencing. The choice of priors was supported by the total as well as real-time monitoring of the rarefaction which indicated sufficient species saturation for both the fungal and as well as bacterial populations across the majority of the samples (Fig. 3 and 4; Fig. S1). The latter also enabled timely decision-making for stopping the run, thereby preserving the flow cell for potential future (re)use. Less than 60% usage of the pores during the entire run of sequencing (30 hours in contrast to the recommended 72-hour runtime), covering both bacteriome and mycobiome in the ~7 million raw and ~5 million high-quality reads at a fully multiplexed (96-sample) scale, was encouraging.

Analysis of the obtained bacteriome identified *S. mitis*, a normal commensal of the human oral cavity (52), as the most dominant bacterial species in the entire population of reads in this study. Previously, microbiome reports, albeit at the genus-level resolution, have consistently reported *Streptococcus* spp. as the primary bacterial genus in the human saliva and oropharynx samples, with *Veilonella* spp., *Neisseria* spp., *Prevotella* spp., *Gemella* spp., *Granulicatella* spp., and *Porphyromonas spp*. being the other frequently reported genera of the oral cavity (8, 33, 53, 54). Consistent with these previous reports, the current study could identify, at a species-level resolution, *N. subflava*, *S. oralis*, *Veilonella parvula*, *Veilonella dispar*, *Granulicatella elegans*, *Streptococcus salivarus*, and *Prevotella melaninogenica* as the other dominant species (Fig. 5; Table S1) following *S. mitis*. These observations, even though specific to Indian subjects, also exhibit a good concordance with the recently characterized core oral microbiota of the European cohort through cost and data intensive whole-genome sequencing (55). The WGS-based taxonomic classification by Caselli and colleagues (55) had identified *S. mitis* as the most abundant bacterial species in the oral cavity along with *S. oralis*, *Haemophilus parainfluenzae*, *P. melaninogenica*, and *N. subflava* as other key microbes of the oral microbiome. This further provides encouraging evidence toward the suitability of achieving species-level resolution for human bacteriome, in concomitance with the mycobiome, in a resource-effective manner through EnsembleSeq.

Simultaneous sequencing of fungal barcoding DNA (ITS region) enabled the potential for useful insights into the mycobiota co-inhabiting the observed bacterial members of the oral microbiome. Among the key fungal taxa, species of *Candida* like, *C. albicans and C. tropicalis*, were consistently observed in this study. *Penicillium spp.* and *Wallemia spp*., along with genera-level assignments for *Aspergillus spp.*, *Cladosporium spp.*, and *Saccharomyces spp.*, were also the major contributors to the total-read population of 0.7 million (Fig. 5; Table S2). While *Candida* species (including *C. albicans*) are regarded as the most common oral cavity-colonizing fungi (14, 36, 56), an oral mycobiota study from the United States (14) has shown that apart from *Candida*, *Malassezia* sp. are another dominant commensal member of the oral mycobiome along with *Aspergilllus*, *Cladosporium*, *Cryptococcus*, *Saccharomyces*, and more. Furthermore, as observed for the bacteriome, the top fungal species (*C. albicans*) identified through the current workflow had good concordance with the only species-level fungal taxon of *C. albicans* reported through WGS by Caselli et al. (55). This highlights the utility of amplicon sequencing in sufficient and rather deep capture of the sparsely abundant members of the rare biosphere (in this case fungi), which could be missed by a data demanding WGS. Members of *Penicillium* and *Wallemia* have also previously been known to be observed in a handful of human oral microbiome (14, 20, 33, 56). Given the understudied and under-characterized nature of the human mycobiota due to the limited available reports, along with a relatively nascent stage of fungal classification databases and algorithms, it is expected that new members may further be identified through future technical advancements.

Contaminants can often bias the observations of microbiome studies. The use of negative control in this study (including pooled negative controls) helped in verifying the minimal overlap between the core oral mycobiota and the fungal species identified in the negative controls, consolidating the reliability of the observations. Moreover, negative control samples had minimal (total 32 reads) contamination of bacterial reads (Fig. 5b and f). It was also encouraging to observe the conformity between the proportions of fungal and bacterial species in positive controls and those obtained in terms of the sequenced reads, indicating the potential of accurate species identification as well as controlling and monitoring the factors that can contribute to the bias in community profiling. It is hoped that our case study and observations, hereby presented as a step-by-step outlined EnsembleSeq workflow, may guide future attempts at further optimizing the right proportion of input molecules, scope of further scale-up, and attempting multiple amplicons (beyond bacteriome and mycobiome) without compromising the expected saturations. We further summarize potential improvements and future directions as the following:

### Lower proportions of fungal spike and going beyond bacteriome and mycobiome

Success in sufficiently capturing the sample-specific mycobiota and bacterial species through a small (25%) spike of fungal ITS DNA in the total library was observed in the current study. This opens the avenue for attempting/optimizing further lower proportions, which may guide the scope for simultaneous spiking of additional kingdom-specific barcoding DNA at the same time. It may be noted that the observed proportion of kingdom-specific reads (e.g., 16S and ITS in our case) may differ from the spiked proportions. We obtained a higher proportion of bacteriome:mycobiome HQ reads (4.7:1 in positive control and 6:1 across all samples) as compared to the original 3:1 proportion used for library preparation. This may be attributed to the availability of (in)sufficient PCR products for one or more of the targeted amplicon types (especially that of sparse fungi) in some clinical samples (Fig. 4a), variation in molar quantifications due to differential lengths of the PCR products, differential proportions of failed or misclassified reads, and human error and other sequencing technology-specific latent factors.

### Multiplexing 16S and ITS region amplification in a single PCR run

In the current research, separate amplification of 16S and ITS region amplification was carried out for all 96 samples. PCR multiplexing is, however, also theoretically possible. Attempts at optimizing the template amount, primer concentrations, and annealing conditions may yield success, thereby further reducing the time and cost associated with multi-amplicon sequencing-based microbiome studies. While this may limit the control over equimolar libraries, post-hoc normalizations can potentially address the skewed library sizes. It may also be noted that factors like choice of primers, sample complexity, and polymerase efficiency can affect the diversity of the captured microbiome, especially of the rare biosphere.

### Toward adaptive barcode prioritization for amplicon (nanopore) sequencing

Real-time access to the DNA sequences enabled continuous monitoring of the species saturation through dynamic classification and rarefaction analysis of the generated reads. With the availability of adaptive sequencing application programming interface (API), this utility of dynamic saturation monitoring may further be extended to barcode prioritization, such that the barcodes that have already saturated for all target microorganisms can be omitted for further sequencing, thereby further optimizing the usage of the sequencing resources. Such implementations of real-time feedback to the sequencing machines may help optimize the use of end-to-end as well as special-purpose computational pipelines frequently employed, post-hoc, for microbiome data processing and analytics (57–59).

### Conclusion

With the advent of high-accuracy long-read sequencing methods, interest has been ignited toward relooking at the human microbiota at a higher resolution. At this juncture, it would be prudent to take strides beyond the traditional approach of treating bacteriome as the “microbiome” and conduct experiments that aim to simultaneously profile different microbial kingdoms of the studied samples. Amplicon sequencing, given its cost effectiveness and ease of data handling, is preferred by microbiome scientists across the world for initial insights and taxonomic characterization of microbial communities. EnsembleSeq workflow, presented in this proof-of-concept report, was attempted with the goal of targeting multiple kingdoms of microorganisms, specifically bacteriome and mycobiome of the total human oral microbiome in a resource- and time-efficient manner. The observations indicated the potential to simultaneously identify both fungal and bacteria species, without compromising on coverage, resolution, or the native community composition, in a single fully multiplexed (96-sample) nanopore amplicon sequencing run. Dynamic tracking of species saturation, enabled by real-time availability of the sequenced DNA, further opens avenues for optimizing the time of run (and even real-time barcode prioritization). Guiding studies are currently needed to add to the confidence of microbiome scientific community that holistic and species-level snapshots of the human microbiome can be achieved without incurring significant costs of time, effort, or resources. We hope to have re-ignited further thought in this direction.

## Data Availability

Abundance data corresponding to the bacteriome and mycobiome of this study are provided in Tables S1 and S2, respectively. Raw sequence data of this study have been submitted to the Open Science Framework (OSF): https://osf.io/h2347/. Real-time base called data has also been submitted to NCBI SRA through Bioproject ID: PRJNA1092079. The passed pod5 files originally generated through the sequencing run were merged using pod5 tools before submission to the OSF. The *in silico* components of the EnsembleSeq workflow are available at: https://github.com/sunilnagpal/EnsembleSeq.

## SUPPLEMENTAL MATERIAL

File S1 spectrum.04150-23-s0001.docx Supplemental methods; Fig. S1 and S2.

Table S1 spectrum.04150-23-s0002.xlsx Species-level bacterial taxonomic assignments.

Table S2 spectrum.04150-23-s0003.xlsx Species-level fungal taxonomic assignments.

ASM does not own the copyrights to Supplemental Material that may be linked to, or accessed through, an article. The authors have granted ASM a non-exclusive, world-wide license to publish the Supplemental Material files. Please contact the corresponding author directly for reuse.
