# Supplementary material for: EnsembleSeq: a workflow towards real-time, rapid, and simultaneous multi-kingdom-amplicon sequencing for holistic and resource-effective microbiome research at scale
Source: Microbiol Spectr. 2024 Apr 30;12(6):e04150-23. doi: 10.1128/spectrum.04150-23 (PMC11237516; doi:10.1128/spectrum.04150-23)
Supplement: File S1 — Supplemental methods; Fig. S1 and S2. [file spectrum.04150-23-s0001.docx]

Supplementary file S1

## EnsembleSeq: A workflow towards real-time, rapid and simultaneous multi-kingdom amplicon sequencing for holistic and resource effective microbiome research at scale

Sunil Nagpal^1,2,3^, Sharmila S. Mande^3^, Harish Hooda^4^, Usha Dutta^4^, Bhupesh Taneja^1,2*^

1. CSIR-Institute of Genomics and Integrative Biology, New Delhi, India
2. Academy of Scientific and Innovative Research (AcSIR), Ghaziabad, India
3. TCS Research, Tata Consultancy Services Ltd, Pune, India
4. Department of Gastroenterology, Post Graduate Institute of Medical Education & Research, Chandigarh, India

*To whom correspondence should be addressed: [btaneja@igib.res.in](mailto:btaneja@igib.res.in)

# Supplementary Methods

## EnsembleSeq positive controls

Briefly, as depicted in Figure 2 (steps 1-3), the genomic DNA of pure cultures of the said strains was extracted using the same DNA extraction process as used for saliva and negative control samples. 16S and ITS amplicons were generated using the extracted genomic DNA as template. These 16S amplicons were quantified using Qubit3.0 and next used as template in 1:4 proportion for the bacteriome (or 2.7:1 for mycobiome) specific positive control sample creation, which was then amplified along with the other samples (Figure 2). The bacteriome and mycobiome specific positive controls so created were also ensembled in 3:1 proportion as performed for other samples (none of the samples was independently processed for library preparation).

## EnsembleSeq library preparation steps

- 1. For each sample, corresponding bacterial and fungal cleaned amplicons were pooled into an approximate 3:1 proportion to make a 133.3 fmol ensembled sample such that it contained 100 fmol of full length 16S rRNA gene (97.4 ng per sample assuming ~1.5kb molecule length) and 33.3 fmol ITS dsDNA molecules (11.9 ng per sample assuming ~550b molecule length). The volume was made upto 7.67 μl using NFW and 0.67 ul of DNA Control Sample (DCS) was added to the pool as available in the [SQK-NBD114.96](https://community.nanoporetech.com/docs/prepare/library_prep_protocols/ligation-sequencing-amplicons-native-barcoding-v14-sqk-nbd114-96/v/nba_9170_v114_revl_15sep2022).
  2. To each of the ensemble sample, 1.67 μl of End-prep master mix (containing 17.55 μl NEB Ultra II End-prep reaction buffer and 7.5 μl Ultra II End-prep Enzyme mix) was added.
  3. All components were thoroughly mixed as per manufacturer’s instructions and incubated in a thermal cycler at 20 ^o^C for 7.5 minutes and 65 ^o^C for 7.5 minutes.
  4. From each of the end-prepped ensemble DNA, 0.75 μl of sample was taken and mixed with 3 μl of NFW followed by 1.25 μl of unique barcode from the 96 barcode plate as available in the native barcoding kit.
  5. The barcodes were ligated to the ensemble DNA using 5 μl of Blunt/TA ligase master mix and incubated at room temperature for 20 minutes.
  6. Each ligation reaction was stopped using 1 μl of EDTA and all barcoded samples were subsequently pooled into two LoBind (Eppendorf) tubes of 1.5 ml each. This yielded ~480 μl of pooled reaction in each LoBind tube.
  7. AMPure XP beads were then added to the pooled reactions at 1.5x volume (i.e. 720 μl) and a standard clean-up was performed as per manufacturer’s instructions (washing twice with 80% molecular grade ethanol). Elution was peformed in 17.5 μl NFW from each tube, totalling 35 μl of total barcoded ensemble-DNA library.
  8. Adapters were ligated to the ensemble-DNA library as instructed in the native barcoding kit:

## Reagent Volume

Pooled barcoded sample 30 µl

Native Adapter (NA) 5 µl NEBNext Quick Ligation Reaction

Buffer (5X)

10 µl

Quick T4 DNA Ligase 5 µl

## Total 50 µl

- 1. Final round of AMPure XP bead based cleaning of adapter ligated library was performed. Instead of alcohol (80% Molecular grade ethanol) washing of beads, this step, as recommended in the native barcoding kit must use 125 μl Short Fragment Buffer (SFB) to avoid downstream damage to the nanopores due to ethanol residues. Additionally, unlike magnetic rack mounted gentle wash, beads should be flicked to resuspend, followed by a spin down, and reracking to the magnetic rack to allow the beads to pellet. Supernatant is subsequently removed using a pipette and stored in a back-up vial for contingency (notably, supernatant can be discarded as recommended by the kit, authors preferred exercising caution and not discarding any supernatant or beads in any of the wash steps of entire protocol to account for potential human errors and hence allowing a chance for recovery of library in case of any contingency). Washing is performed twice as instructed. Elution was performed by resuspending the washed pellet in 15 µl Elution Buffer (EB).
  2. Washed, adapter ligated ensemble-DNA library was quantified using Qubit 3.0. 20ng of this library was used to make the final sequencing library:

Reagent Volume per flow cell

Sequencing Buffer (SB) 37.5 µl Library Beads (LIB) mixed immediately before

use

25.5 µl

DNA library 12 µl (containing 20ng in EB)

## Total 75 µl

- 1. The above final library was loaded into the primed R10.4.1 flow cell (FLO-MIN114) mounted in an mk1c machine containing the MinKNOW version 23.04.5 as instructed in the [Ligation sequencing amplicons - Native Barcoding Kit 96 V14 (SQK-NBD114.96) protocol](https://community.nanoporetech.com/docs/prepare/library_prep_protocols/ligation-sequencing-amplicons-native-barcoding-v14-sqk-nbd114-96/v/nba_9170_v114_revl_15sep2022).

# Figure S1


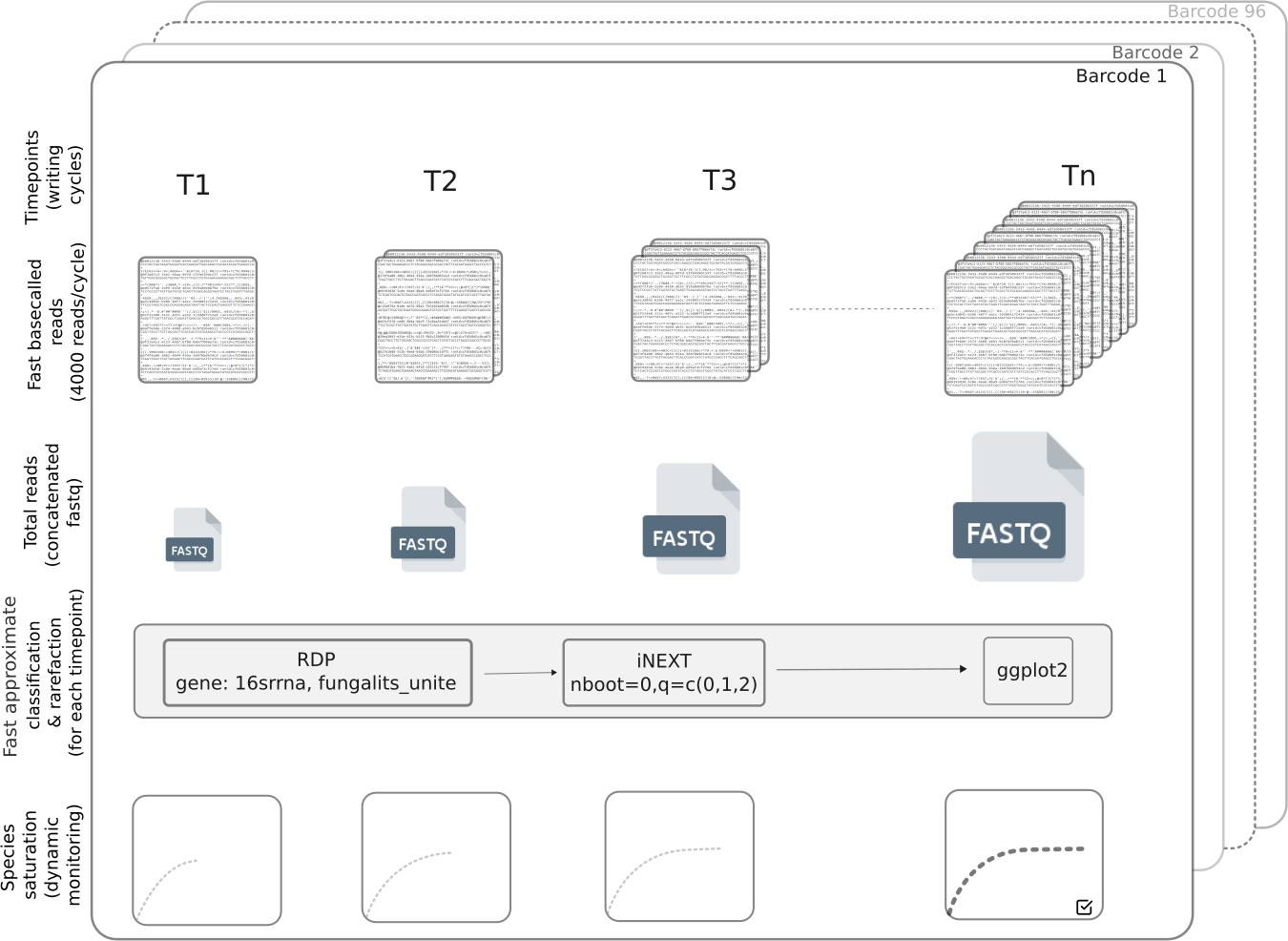


**Figure S1.** Schematic representation of the dynamic monitoring of species saturation that can guide the optimal run-time for nanopore sequencing of ensembled amplicons. T1,T2,T3...Tn represent various time points after starting the sequencing run, where T1 refers to the time when first set of sequences are real-time base called and written on the disc. Subsequent time points refer to the events of new sequences being written on disc (for e.g. nanopore guppy basecaller v6.5.7+ca6d6afb8 is configured to write 4000 reads in each writing cycle by default). For each time point, all available reads can be concatenated to a single fastq file and the reads can be subjected to direct taxonomic classification (fast but approximate) using RDP classifier (v2.14), employing both bacterial and fungal databases. The accumulated classified sequences can then be separately analysed for species saturation through rarefaction analysis (using iNEXT (v3.0.0) library of R programming language) and the rarefaction plot for the given time point can be plotted using ggplot2 (v3.4.4) library of R. Taxonomic classifications of independent time point specific sequences (without sequence accumulation) can also be aggregated to the accumulated taxonomic composition of the previous time point to yied the similar temporal trend of rarefaction. The dynamic monitoring step of EnsembleSeq workflow is coined Raredynamics (refer EnsembleSeq in-silico workflow/codebase).

# Figure S2


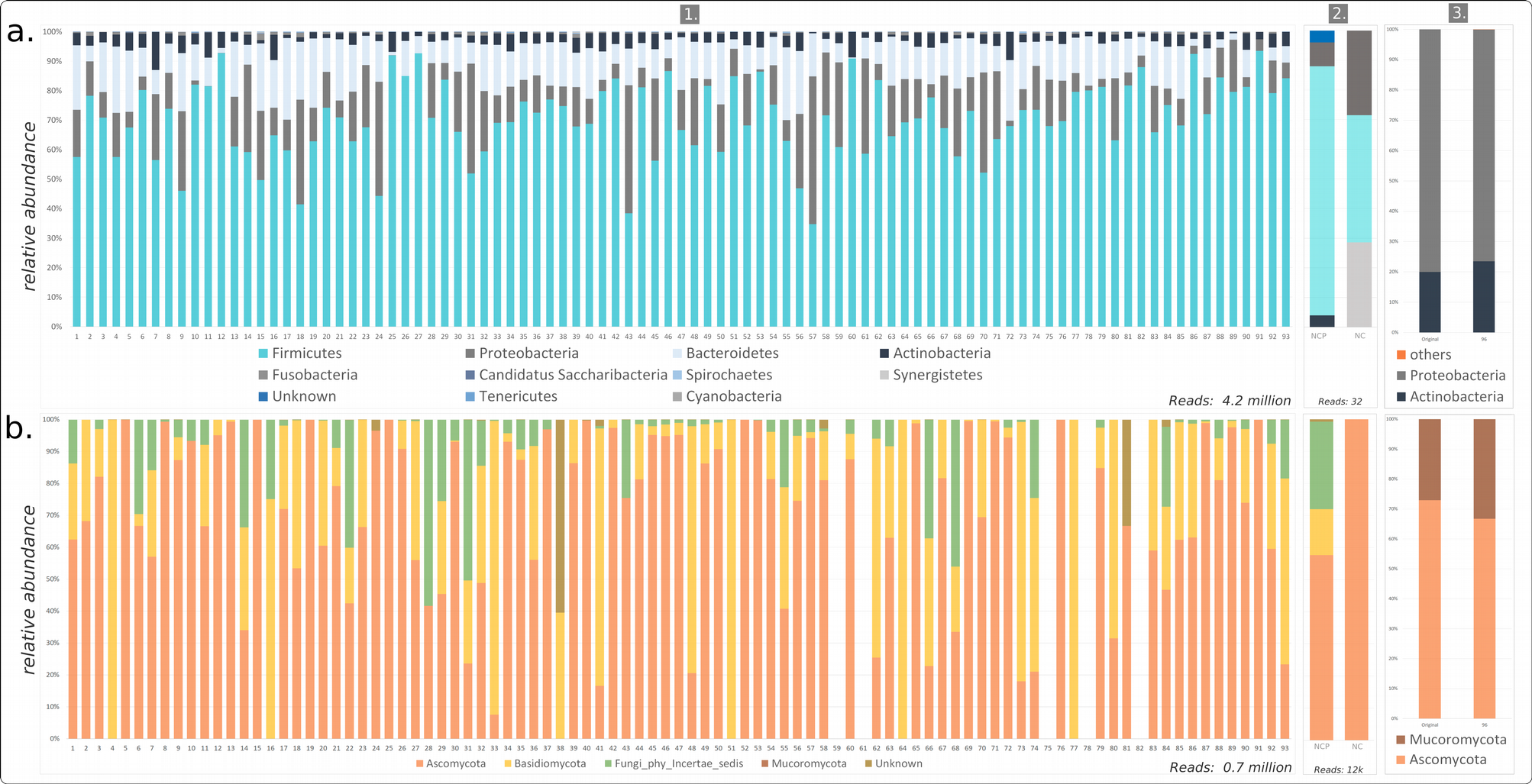


**Figure S2**. Relative abundance of high-quality assigned reads in the samples of this study at phylum level of taxonomic hierarchy for (a) bacteriome and (b) mycobiome. Assignment for negative control samples (NCP and NC) are provided in a2 and b2 while for positive control samples is provided in a3 and b3. Original intended proportion of the positive control molecules are provided in the left stack bar of a3 and b3 panels.
